# Sarcopenia is associated with osteopenia and impaired quality of life in children with genetic intrahepatic cholestatic liver disease

**DOI:** 10.1097/HC9.0000000000000293

**Published:** 2023-10-31

**Authors:** Julia M. Boster, Nathan P. Goodrich, Cathie Spino, Kathleen M. Loomes, Estella M. Alonso, Binita M. Kamath, Ronald J. Sokol, Saul Karpen, Alexander Miethke, Benjamin L. Shneider, Jean P. Molleston, Rohit Kohli, Simon P. Horslen, Philip Rosenthal, Pamela L. Valentino, Jeffrey H. Teckman, Thomas N. Hangartner, Shikha S. Sundaram

**Affiliations:** 1 Department of Pediatrics, Pediatric Liver Center, Digestive Health Institute and Section of Pediatric Gastroenterology, Hepatology & Nutrition, Children’s Hospital Colorado, University of Colorado School of Medicine, Aurora, Colorado, USA; 2 Arbor Research Collaborative for Health, Ann Arbor, Michigan, USA; 3 Department of Biostatistics, University of Michigan, Ann Arbor, Michigan, USA; 4 Division of Gastroenterology, Hepatology and Nutrition, The Children’s Hospital of Philadelphia, Philadelphia, Pennsylvania, USA; 5 Ann and Robert Lurie Children’s Hospital, Chicago, Illinois, USA; 6 Division of Gastroenterology, Hepatology and Nutrition, Hospital for Sick Children, University of Toronto, Toronto, Ontario, Canada; 7 Children’s Healthcare of Atlanta, Atlanta, Georgia, USA; 8 Cincinnati Children’s Hospital Medical, Cincinnati, Ohio, USA; 9 Baylor College of Medicine, Texas Children’s Hospital, Houston, Texas, USA; 10 Riley Hospital for Children, Indianapolis, Indiana, USA; 11 Children’s Hospital Los Angeles, Los Angeles, California, USA; 12 UPMC Children’s Hospital of Pittsburgh, Pittsburgh, Pennsylvania, USA; 13 UCSF Benioff Children’s Hospital, San Francisco, California, USA; 14 Seattle Children’s Hospital, Seattle, Washington, USA; 15 Saint Louis University School of Medicine, St. Louis, Missouri, USA; 16 Department of Biomedical, Industrial & Human Factors Engineering, Wright State University, Dayton, Ohio, USA

## Abstract

**Background:**

Sarcopenia occurs in pediatric chronic liver disease, although the prevalence and contributing factors in genetic intrahepatic cholestasis are not well-described. The objective of this study was to measure muscle mass in school-aged children with genetic intrahepatic cholestasis and assess relationships between sarcopenia, clinical variables, and outcomes.

**Methods:**

Estimated skeletal muscle mass (eSMM) was calculated on dual-energy x-ray absorptiometry obtained in a Childhood Liver Disease Research Network study of children with bile acid synthesis disorders(BASD) alpha-1 antitrypsin deficiency (a1ATd), chronic intrahepatic cholestasis (CIC), and Alagille syndrome (ALGS). Relationships between eSMM, liver disease, and transplant-free survival were assessed.

**Results:**

eSMM was calculated in 127 participants (5–18 y): 12 BASD, 41 a1ATd, 33 CIC, and 41 ALGS. eSMM z-score was lower in CIC (−1.6 ± 1.3) and ALGS (−2.1 ± 1.0) than BASD (-0.1 ± 1.1) and a1ATd (−0.5 ± 0.8, *p* < 0.001). Sarcopenia (defined as eSMM z-score ≤− 2) was present in 33.3% of CIC and 41.5% of ALGS participants. eSMM correlated with bone mineral density in the 4 disease groups (*r*=0.52–0.55, *p* < 0.001–0.07), but not serum bile acids, bilirubin, aspartate aminotransferase/platelet ratio index, or clinically evident portal hypertension. Of the 2 patients who died (1 with sarcopenia) and 18 who underwent liver transplant (LT, 4 with sarcopenia), eSMM z-score did not predict transplant-free survival. eSMM z-score correlated with the Physical Pediatric Quality of Life Inventory score (*r*=0.38–0.53, *p* = 0.007–0.04) in CIC and a1ATd.

**Conclusion:**

Severe sarcopenia occurs in some children with ALGS and CIC. The lack of correlation between eSMM and biochemical cholestasis suggests mechanisms beyond cholestasis contribute to sarcopenia. While sarcopenia did not predict transplant-free survival, LT and death were infrequent events. Future studies may define mechanisms of sarcopenia in genetic intrahepatic cholestasis.

## INTRODUCTION

Sarcopenia, defined by impairments in skeletal muscle mass and function, is highly prevalent in adults with chronic liver disease and has been associated with profound negative effects on patient outcomes.^1–6^ Although pediatric data are limited, results demonstrating muscle wasting in children are emerging across the age span and within various underlying causes of chronic liver disease.^7–15^ Most of the pediatric sarcopenia literature includes a heterogenous group of liver diseases, and no study has specifically assessed muscle mass in children with genetic intrahepatic cholestastic diseases, such as bile acid synthesis disorders (BASD), alpha-1 antitrypsin deficiency (a1ATd), progressive familial intrahepatic cholestasis (PFIC), and Alagille syndrome (ALGS). However, other alterations in body composition, including impairments in linear growth and bone mineral density (BMD), are well-established in this group of diseases.^16^

The implications of muscle wasting in children likely differ from those in adults with liver disease, as body composition and muscle function, particularly as it relates to psychomotor development, vary greatly across the age span. Additionally, the mechanisms driving sarcopenia in children with liver disease likely differ from those in adults, given the vastly different underlying etiologies that lead to chronic liver disease in children, such as genetic cholestatic diseases. Acknowledging the other alterations in body composition that occur in these diseases and the evolving understanding of sarcopenia as an important clinical entity in children with liver disease, we aimed to assess for sarcopenia in children with genetic intrahepatic cholestasis and to examine the relationship between muscle mass, key clinical variables, and patient outcomes. We also explored the relationship of muscle mass to health-related quality of life (QOL), given the known association between sarcopenia and QOL in adults with chronic liver disease.^17^

## METHODS

### Study design and participants

Research participants were enrolled through the Longitudinal Study of Genetic Causes of Intrahepatic Cholestasis (LOGIC) protocol (NCT00571272). The LOGIC protocol is an observational, natural history study of children with genetic causes of intrahepatic cholestasis organized within the Childhood Liver Disease Research Network, a consortium funded by the National Institutes of Health’s National Institute of Diabetes and Digestive and Kidney Diseases encompassing 14 pediatric academic centers across North America. Participants with BASD, a1ATd, ALGS, and PFIC are enrolled from birth to age 25 years. Centralized research genetic testing was not performed for all clinical PFIC participants in this cohort; therefore, we herein classify this group as “chronic intrahepatic cholestasis (CIC)” for the purpose of this study. The diagnosis of ALGS was established by either clinical criteria or genetic testing (*JAGGED1* or *NOTCH2*), a1ATd by protease inhibitor typing or genetic testing (*SERPINA1*), and BASD by urine bile acid composition or genetic testing, all as described.^16^

The LOGIC protocol prospectively collects data elements, including laboratory values, growth parameters, physical examination findings, and clinical events at yearly intervals for up to 20 years or until death, liver transplant (LT), or study dropout. LOGIC participants ≥ 5 years old with their native liver were eligible to undergo dual-energy x-ray absorptiometry (DEXA) once during the study period (2008–2013). DEXA scans were analyzed for bone mineral density in children with genetic intrahepatic cholestasis and reported by Loomes et al in 2019.^16^ These same DEXA scans were analyzed for the present study, as described below, to determine the estimated skeletal muscle mass (eSMM). All research was conducted in accordance with both the Declarations of Helsinki and Istanbul. The study was approved by institutional review boards at each center. Written informed consent was obtained from parents/guardians or participants 18 years or older and assent from children > 7 years of age.

### DEXA-based measurements

Research DEXA scans were performed on Hologic (Newark, DE) or Lunar (GE Health Sciences, Pittsburgh, PA) equipment and analyzed to measure appendicular lean mass (ALM), the sum of bilateral upper and lower extremity lean mass. ALM has been shown to accurately predict whole-body MRI-measured total SMM in children when using the following equations, based on Tanner stage of the child—Tanner stage 5: eSMM (kg) = (1.19 × ALM (kg)) – 1.65; Tanner stage <5: eSMM (kg) = (1.115 × ALM (kg)) – 1.135.^18^ Tanner stage was determined by physical exam, participant or parent report and available in 85% of participants. In the 15% who were missing these data, Tanner stage was presumed to be 5 in children 15 years of age or older, based on population data indicating that the majority of children achieve Tanner stage 5 by 15 years of age).^19^

An eSMM z-score was calculated for each participant based on a comparison to normative population data.^20^ DEXA is a useful modality for studying sarcopenia in children, and was thus utilized in this study due to (1) its ability to estimate total SMM across the age span; (2) minimal radiation dose (as opposed to CT scan); and (3) relative ease and speed of scans, allowing younger children to participate without the need for sedation.

BMD was determined on DEXA scans of the whole body, spine, and femur. Scans were standardized using BioMedical Imaging Laboratory phantoms provided by the DEXA Coordinating Center, and adjustments between the Lunar and Hologic scanners were performed with patient-based correction formulas.^16^ BMD z-scores were calculated based on age, sex, and race reference data.^21^

### Quality of life measurements

The Pediatric Quality of Life Inventory (PedsQL) 4.0 was used to measure health-related QOL scores in 4 domains: Physical Function, Emotional Functioning, Social Functioning, and School Functioning. As a surrogate assessment for the functional status of each participant, the Total and Physical QOL scores were assessed in relation to eSMM. While the PedsQL score is not a direct measure of muscle function, it has been shown to correlate with hand-grip strength in other diseases.^22^

### Statistical analysis

Differences in continuous variables were tested with Wilcoxon rank-sum tests for 2 groups or Kruskal-Wallis tests for more than 2 groups. When indicated, pairwise multiple comparisons testing was performed using the Dwass, Steel, Critchlow-Fligner method. Linear regression was used to test differences in eSMM z-score by diagnosis while adjusting for total bilirubin, serum bile acids, albumin, and aspartate aminotransferase (AST) to platelet ratio index (APRI). Differences in categorical variables were tested with chi-Square tests. The relationships between eSMM z-score and biochemical markers of liver disease, as well as BMD, were assessed visually using scatter plots with locally estimated scatterplot smoothing (Loess) lines and quantitatively by Spearman’s correlation. Sarcopenia was defined as an eSMM z-score ≤ − 2; its effect on transplant-free survival (TFS) was assessed by Kaplan-Meier analysis, while the effect of eSMM z-score (as a continuous variable, controlling for height) on TFS was studied with Cox regression analysis. Linear regression analysis was used to assess the relationship between clinically evident portal hypertension (CEPH) and sarcopenia. We included both definite CEPH (clinical findings of splenomegaly and thrombocytopenia or manifestations of ascites or varices seen endoscopically) and possible CEPH (either splenomegaly or thrombocytopenia without ascites or varices), as defined in prior Childhood Liver Disease Research Network studies.^23^ Finally, Spearman’s correlation was used to study the relationship between SMM and health-related QOL, as measured by the PedsQL4.0 tool. Correlation and survival analyses were performed separately for each liver disease to account for the confounding effect of underlying disease process on SMM.

## RESULTS

DEXA scans were performed in 127 children with genetic intrahepatic cholestasis—12 with BASD, 41 with a1ATd, 33 with CIC, and 41 with ALGS. While all patients classified as CIC met clinical criteria for enrollment in LOGIC, 19 of 33 (58%) had confirmatory research genetic testing: 8 had mutations in adenosine triphosphatase phospholipid transporting 8B1 (familial intrahepatic cholestasis 1 deficiency), 7 had mutations in adenosine triphosphate-binding cassette subfamily B member 11 ( bile salt export pump deficiency), and 4 had mutations in ATP-binding cassette subfamily B *4* (multidrug resistance deficiency).

Table 1 displays the demographic and clinical features of study participants by diagnosis. Age, sex, race, and ethnicity were similar across diseases. Height, weight, and body mass index were significantly different across diseases (*p* < 0.01) and lowest in CIC and ALGS. ALGS participants had significantly higher total and direct bilirubin, gamma-glutamyl transpeptidase (GGT), AST, and APRI (*p* < 0.001). Serum bile acids were lowest in BASD (mean 26.9 ± 47.5 μmol/L), but still higher than expected in a disease characterized by low serum bile acids. After removing the one extreme outlier with an implausible bile acid measurement of 161.1 μmol/L, the mean serum bile acids in BASD participants were 12.0 ± 5.7 μmol/L (Table 1).

**TABLE 1:** Demographic and clinical characteristics by disease

	BASD (n=12)	a1ATd (n=41)	CIC (n=33)	ALGS (n=41)	*p* ^a^
Demographic
Age (y)^b^	10.2 (5.4)	10.2 (4.6)	11.2 (4.7)	9.8 (3.5)	0.72
Sex, n (%)					
Female	3 (25.0)	12 (29.3)	17 (51.5)	18 (43.9)	0.16
Male	9 (75.0)	29 (70.7)	16 (48.5)	25 (56.1)	—
Race, n (%)					
Asian	1 (8.3)	—	1 (3.0)	—	0.22
Black or African	1 (8.3)	—	2 (6.1)	4 (9.8)	—
American	9 (75.0)	40 (97.6)	27 (81.8)	32 (78.0)	—
White	1 (8.3)	1 (2.4)	1 (3.0)	3 (7.3)	—
Other or Multiracial Unknown	—	—	2 (6.1)	2 (4.9)	—
Ethnicity, n (%)					
Hispanic/Latino	3 (25.0)	3 (7.3)	7 (21.2)	3 (7.3)	0.33
Non-Hispanic/Latino	9 (75.0)	37 (90.2)	26 (78.8)	37 (90.2)	—
Unknown	—	1 (2.4)	—	1 (2.4)	—
Clinical Characteristic^b^					
Height z-score	−0.2 (0.7) (n = 11)	0.5 (1.2) (n = 39)	−1.2 (1.8) (n = 33)	−1.7 (1.0) (n = 41)	**<0.001**
Weight z-score	0.5 (1.1) (n = 11)	0.5 (0.9) (n = 39)	−1.0 (1.6) (n = 33)	−1.6 (1.3) (n = 41)	**<0.001**
BMI (kg/m^2^)	19.9 (5.1) (n = 12)	18.6 (3.9) (n = 41)	17.7 (3.7) (n = 33)	16.1 (2.1) (n = 41)	**0.004**
Albumin (g/dL)	4.6 (0.3) (n = 12)	4.4 (0.4) (n = 41)	4.2 (0.5) (n = 33)	4.2 (0.6) (n = 40)	**0.02**
INR	1.0 (0.1) (n = 12)	1.1 (0.1) (n = 37)	1.1 (0.2) (n = 28)	1.1 (0.2) (n = 38)	0.55
Total bilirubin (mg/dL)	0.4 (0.3) (n = 12)	0.6 (0.7) (n = 41)	1.4 (1.8) (n = 33)	3.8 (5.2) (n = 40)	**<0.001**
Direct bilirubin (mg/dL)	—	(0.1) (n=21)	0.3 (0.6) (n = 18)	3.2 (3.4) (n = 16)	**<0.001**
GGT (IU/L)	23.6 (4.9) (n = 10)	57.5 (68.6) (n = 37)	85.0 (163.4) (n = 31)	337.7 (302.6) (n = 33)	**<0.001**
Bile acids (μmol/L)	12.0 (5.7) (n = 9)^c^	16.7 (19.6) (n = 35)	78.0 (112.7) (n = 26)	131.7 (111.5) (n = 32)	**<0.001**
AST (IU/L)	54.1 (17.1) (n = 12)	62.3 (49.2) (n = 41)	73.8 (43.1) (n = 33)	161.3 (114.3) (n = 40)	**<0.001**
Platelet count (10^3^/mm)	269 (105) (n = 12)	238 (114) (n = 41)	296 (139) (n = 30)	238 (109) (n = 38)	0.28
APRI	0.6 (0.4) (n = 12)	(1.9) (n = 41)	0.9 (1.1) (n = 30)	2.5 (2.4) (n = 38)	**<0.001**
Established CEPH prior to DEXA (n (%))	3 (25)	9 (22)	5 (15)	18 (44)	**0.03**
PedsQL Total (participant)	88.3 (6.8) (n = 7)	76.9 (16.3) (n = 33)	72.4 (17.6) (n = 25)	73.7 (13.2) (n = 29)	0.07
PedsQL Total (parent)	83.9 (13.4) (n = 9)	84.4 (16.1) (n = 34)	77.4 (13.3) (n = 27)	71.5 (16.1) (n = 31)	**0.006**
PedsQL Physical (participant)	89.3 (9.2) (n = 7)	80.6 (16.2) (n = 34)	77.9 (19.5) (n = 25)	80.5 (15.3) (n = 29)	0.60
PedsQL Physical (parent)	92.7 (5.2) (n = 9)	87.7 (18.4) (n = 34)	79.7 (14.7) (n = 27)	71.7 (20.4) (n = 30)	**<0.001**

a Differences among diagnosis groups tested with Kruskal-Wallis tests for continuous variables and chi-square tests for categorical variables.

b Results expressed as mean (SD), unless otherwise specified.

c One extreme outlier with the value of 161.1 μmol/L was removed in this analysis.

Note: Bolded items indicate p-values that are statistically significant at ≤ 0.05.

Abbreviations: a1ATd, Alpha-1 antitrypsin deficiency; ALGS, Alagille syndrome; APRI, AST to platelet ratio index; AST, aspartate aminotransferase; BASD, bile acid synthesis disorders; BMI, body mass index; CEPH, clinically evident portal hypertension; CIC, chronic intrahepatic cholestasis; DEXA, dual-energy x-ray absorptiometry; eSMM, estimated skeletal muscle mass; GGT, gamma-glutamyl transpeptidase; INR, international normalized ratio; PedsQL, Pediatric Quality of Life Inventory.

**FIGURE 1:**
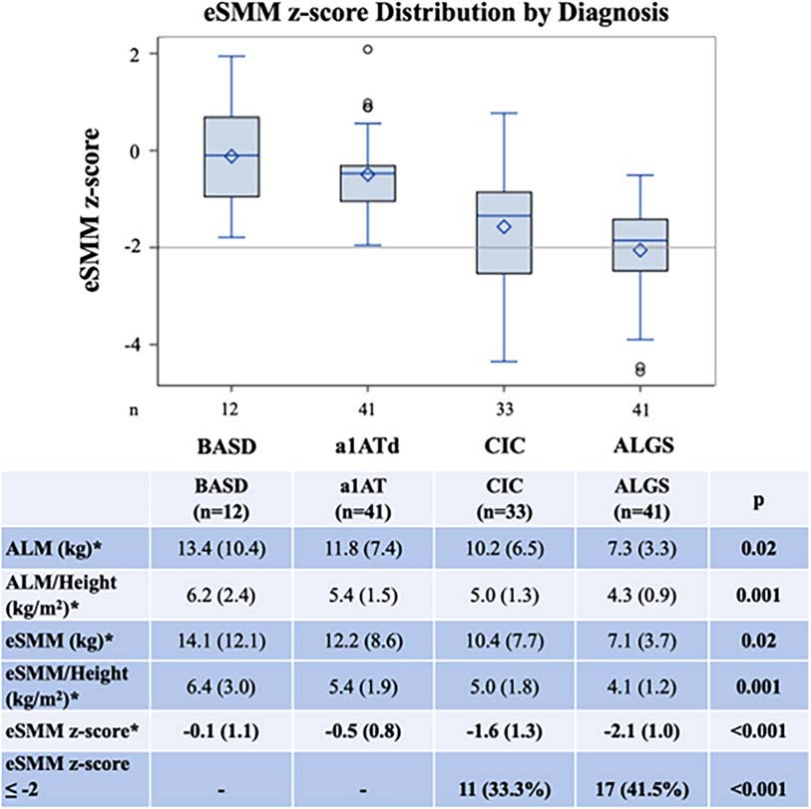
Distribution of Muscle Mass by Underlying Cholestatic Liver Disease. * Results expressed as Mean (SD). Abbreviations: a1ATd, alpha-1 antitrypsin deficiency; ALGS, Alagille syndrome; ALM, appendicular lean mass; BASD, bile acid synthesis disorders; CIC, chronic intrahepatic cholestasis; eSMM, Estimated skeletal muscle mass.

eSMM was significantly lower in CIC and ALGS than BASD and a1ATd and compared with normative population data. Participants with ALGS demonstrated the lowest eSMM (Figure 1). Mean ALM and eSMM were significantly different among diagnosis groups, even after adjusting these measurements to participant height. The eSMM z-score (accounting for age and sex) was also significantly different by diagnosis (*p* < 0.001). Pairwise testing for eSMM z-score adjusted for multiple comparisons showed that CIC (−1.6 ± 1.3) and ALGS (−2.1 ± 1.0) were similar to each other and significantly lower than BASD (−0.1 ± 1.1) and a1ATd (−0.5 ± 0.8) (*p* < 0.001). The difference in the eSMM z-score between diagnoses remained significant after adjusting for total bilirubin, total bile acids, albumin, and APRI (overall *p* < 0.001, pairwise CIC vs. BASD, CIC vs. a1ATd, ALGS vs. BASD, and ALGS vs. a1ATd all *p* < 0.002). Of note, 33.3% of CIC participants and 41.5% of ALGS participants had an eSMM z-score ≤− 2 (Figure 1).

The eSMM z-score strongly correlated with the BMD z-score in all diseases (*r* = 0.52–0.55, *p* < 0.001–0.07; Table 2). Not surprisingly, the eSMM z-score and height z-score had a strong correlation, representing higher eSMM in taller participants. As such, height was included in subsequent models to ensure it was not acting as a confounder. Body mass index z-score moderately correlated with the eSMM z-score across diseases (Supplemental Table 1, http://links.lww.com/HC9/A600).

**TABLE 2:** Spearman correlations by disease

Correlation	Disease	Spearman *r*	Spearman *p*
eSMM z-score and whole body BMD	BASD	0.55	0.07
	CIC	0.55	**<0.001**
	a1ATd	0.52	**<0.001**
	ALGS	0.54	**<0.001**
eSMM z-score and serum bile acids (μmol/L)	BASD	0.03	0.93
	CIC	−0.21	0.31
	a1ATd	−0.04	0.80
	ALGS	−0.35	0.05
eSMM z-score and total bilirubin (mg/dL)	BASD	−0.03	0.34
	CIC	−0.14	0.43
	a1ATd	0.16	0.33
	ALGS	−0.24	0.13
eSMM z-score and participant-reported *Total* PedsQL score	BASD	−0.61	0.15
	CIC	0.54	**0.005**
	a1ATd	0.39	0.03
	ALGS	0.06	0.76
eSMM z-score and parent-reported *Total* PedsQL score	BASD	−0.45	0.22
	CIC	0.37	0.06
	a1ATd	0.28	0.11
	ALGS	−0.16	0.38
eSMM z-score and participant-reported *Physical* PedsQL score	BASD	−0.47	0.29
	CIC	0.53	**0.007**
	a1ATd	0.42	**0.01**
	ALGS	−0.05	0.08
eSMM z-score and parent-reported *Physical* PedsQL score	BASD	−0.34	0.37
	CIC	0.39	**0.04**
	a1ATd	0.38	**0.03**
	ALGS	−0.16	0.39

Note: Bolded items indicate p-values that are statistically significant at ≤ 0.05.

Abbreviations: a1ATd, Alpha-1 antitrypsin deficiency; ALGS, Alagille syndrome; eSMM, BASD, bile acid synthesis disorders; BMD, bone mineral density; CIC, chronic intrahepatic cholestasis; estimated skeletal muscle mass; PedsQL, Pediatric Quality of Life Inventory.

eSMM was not significantly correlated with the biochemical markers of cholestasis. With the exception of a weak inverse relationship between eSMM z-score and serum bile acids in ALGS (r=-0.35, *p*=0.05), there were no significant correlations between eSMM z-score and serum bile acids (Table 2), total bilirubin (Table 2), or GGT (Supplemental Table 1, http://links.lww.com/HC9/A600) across diseases. Biliary diversion was performed in 17 children with CIC and 8 with ALGS before the DEXA scan. The mean eSMM z-score did not differ between those with or without biliary diversion (−1.9 ± 1.4 vs. −1.2 ± 1.1 in CIC; −1.8 ± 0.6 vs. −2.1 ± 1.1 in ALGS; *p* ≥ 0.11). In addition, we did not find notable correlations between eSMM z-score and albumin, AST, platelet count, or APRI (Supplemental Table 1, http://links.lww.com/HC9/A600). The above Spearman correlations were also performed using ALM standardized to height (kg/m^2^) as the muscle mass variable, as is typical in DEXA-based adult sarcopenia literature, and the results were comparable.

A total of 35 children had definite or possible CEPH before undergoing DEXA (3 BASD, 9 a1ATd, 5 CIC, and 18 ALGS participants), and 17 developed CEPH in the follow-up period after DEXA (2 a1ATd, 7 CIC, and 8 ALGS). The eSMM z-score was not significantly different in any disease between children with or without established CEPH at the time of DEXA (difference in the eSMM z-score ranging from −0.52 to 0.66 between those with and without CEPH, all *p* > 0.41). Similarly, the presence or absence of CEPH did not predict a lower eSMM z-score when studied across all diseases together but controlled for underlying disease in a linear regression model (estimate = 0.07, *p* = 0.75). There were too few children with sarcopenia (eSMM z-score ≤− 2) who developed the new onset of CEPH in the follow-up period after DEXA to test the effect of sarcopenia on subsequent CEPH development (Table 3).

**TABLE 3:** Event number by disease and sarcopenia status

	Events post-DEXA (all participants)/events in participants with sarcopenia^a^			
Outcome	BASD	a1ATd	CIC	ALGS
Death	0/−	0/−	0/0	2/1
Liver transplant	0/−	3/−	9/2	9/2
Onset of CEPH	0/−	2/−	7/2	8/2
Variceal bleed	0/−	2/−	1/0	1/0
Onset of ascites	0/−	1/−	3/1	7/3
Fracture	1/−	4/−	5/2	11/5
Composite event^b^	1/−	6/−	13/4	17/7

a Sarcopenia as defined by an eSMM z-score ≤− 2. (No subjects with a1ATd or BASD had an eSMM z-score ≤− 2, so these fields are left blank).

b Composite event: death, transplant, variceal bleed, onset of ascites, or bone fracture.

Abbreviations: a1ATd, Alpha-1 antitrypsin deficiency; ALGS, Alagille syndrome; BASD, bile acid synthesis disorders; CEPH, clinically evident portal hypertension. CIC, chronic intrahepatic cholestasis; DEXA, dual-energy x-ray absorptiometry.

The eSMM z-score did not affect TFS by Kaplan-Meier (Log-rank *p* = 0.82 in CIC, *p* = 0.38 in ALGS) or Cox Regression analysis *(CIC HR 0.71, 95% CI [0.21, 2.44], p = 0.59; ALGS HR 1.29, 95% CI [0.60, 2.78], p = 0.52)*. Time-to-event analyses were not conducted for BASD or a1ATd due to the insufficient number of events. There were limited numbers of children with sarcopenia who experienced death or transplant within each disease process (Table 3), limiting the power to detect potential differences in TFS in this cohort. When studying CIC and ALGS as a combined group, the 2 diseases in which participants had eSMM z-scores ≤− 2, sarcopenia did not affect the composite outcome of death, transplant, variceal bleed, onset of ascites, or bone fracture (*p* = 0.58) (Supplemental Figure 1, http://links.lww.com/HC9/A601).

The parent-reported Total PedsQL and Physical PedsQL score were lowest in ALGS (mean Total score 71.5 ± 16.1, mean Physical score 71.7 ± 20.1 (*p* = 0.006 and < 0.001)). Participant-reported PedsQL scores (Total and Physical domains) were similar across diseases (Table 1). The eSMM z-score had a moderate correlation with participant-reported Total PedsQL in CIC and a1ATd (*r* = 0.54 and 0.39, *p* = 0.005 and 0.03) but not in parent-reported Total PedsQL score (Table 2). Also, in CIC and a1ATd, the eSMM z-score correlated with both participant-reported Physical PedsQL (CIC: *r* = 0.53, *p* = 0.007; a1ATd: *r* = 0.42, *p* = 0.01) and parent-reported Physical PedsQL scores (CIC: *r* = 0.39, *p* = 0.04; a1ATd: *r* = 0.38, *p* = 0.03) (Table 2).

The parent-child agreement in Total and Physical PedsQL scores was studied in 104-paired parent and child reports using intra-class correlation (ICC). ICC was relatively low across diseases in both Total (ICC = 0.36) and Physical (ICC = 0.41) scores (Supplemental Table 2, http://links.lww.com/HC9/A600).

## DISCUSSION

In this cohort of school-aged children with genetic intrahepatic cholestasis, CIC and ALGS patients had significant sarcopenia, with ~one-third of CIC participants and nearly half of ALGS participants demonstrating an eSMM z-score that was at least 2 SDs below population means. The difference in muscle mass between diseases was significant, even after adjusting for height, suggesting that stunting of linear growth does not fully explain the loss of muscle mass in children with genetic intrahepatic cholestasis.

This study only included children with BASD, a1ATd, CIC, and ALGS who were ≥ 5 years of age with their native liver. Otherwise stated, all participants had survived to age 5 years without requiring an LT, selecting a group of children with less severe cholestatic liver disease in early childhood, highlighted by the ALGS group. In long-term follow-up of children with ALGS-associated cholestasis, between 60% and 76% require LT or die by adulthood.^24,25^ In the subset of LOGIC participants we studied with DEXA, only 9 of 41 (22%) underwent LT in longitudinal follow-up, thus likely representing a group with less severe manifestations of cholestasis than the general ALGS population. Still, muscle mass was severely affected in children with ALGS and CIC despite being a relatively “healthier” group from a cholestatic liver disease standpoint.

This study identified a strong correlation between eSMM and BMD in children with genetic intrahepatic cholestasis. Although this is a novel finding in this disease group, the overlap between sarcopenia and osteopenia (known as “osteosarcopenia”) is well-described in aging and other disease states.^26–29^ In the older-age adult population and adults with chronic liver disease, the cumulative effects of osteopenia and sarcopenia lead to a higher risk of falls and fractures as well as higher health care cost and impairments in QOL, as compared with each entity separately.^26,27^ Osteosarcopenia has been increasingly recognized in other pediatric diseases, such as inflammatory bowel disease and pediatric obesity.^29,30^

Given the lifelong cross-talk that occurs between the bone and muscle, mechanisms leading to impaired BMD and the loss of muscle mass likely overlap in many disease states, involving a combination of endocrine and paracrine dysfunction and increased inflammatory cytokine activity.^31,32^ The effect of cholestasis on bone composition was explored in the same cohort of children with genetic intrahepatic cholestasis studied herein, revealing an inverse correlation between biochemical markers of cholestasis and BMD, specifically in ALGS (bilirubin and total hip BMD: *r*=− 0.66; serum bile acids and total hip BMD: *r*=− 0.36).^16^ The investigators hypothesized that cholestasis has direct effects on bone metabolism, citing in vitro and in vivo studies that support this hypothesis (including one in which human osteoblasts treated with bilirubin and sera from jaundiced patients showed decreased viability and differentiation).^33^ In the current study, we did not observe a correlation between biochemical cholestasis and eSMM, indicating that cholestasis may not be the primary driver of muscle wasting in children with genetic cholestasis. The strong correlation between eSMM and BMD does, however, argue for overlapping mechanisms in sarcopenia and osteopenia, although more rigorous mechanistic studies are needed.

Similarly, we did not detect a correlation between eSMM and the markers of liver disease severity captured in this study (albumin, INR, AST, platelet count, and APRI). The markers used to assess the “degree of cholestasis” and “disease severity” have notable limitations, however. Serum bilirubin may not adequately represent the degree of cholestasis, and GGT has limitations in that some of these diseases are typified by low GGT (eg, BASD, FIC1, and bile salt export pump deficiency). Data were unavailable for the period of time between biliary diversion and DEXA. Based on this limitation and the relatively low number of biliary diversions, we are unable to ascertain the impact of surgery on the relationship between serum bile acid and muscle mass. Additionally, we acknowledge the heterogenous genetic basis of the CIC and BASD groups, but the limited number of children with individual genetic subtypes was too small to make any meaningful comparisons. Perhaps most importantly, this is a cohort of children with less severe cholestasis (and presumably with less severe clinical manifestations of cholestasis) in that they have survived to age 5 years without requiring a transplant. Thus, while the data indicate a lack of correlation between muscle mass and cholestasis, this cannot be concluded with certainty in all children with genetic cholestatic liver disease.

Additionally, while platelet count and APRI may function as noninvasive assessments of portal hypertension and surrogate markers of hepatic fibrosis, they are certainly not as sensitive as liver biopsy, which was not obtained in this study. As such, the correlation between the degree of hepatic fibrosis and eSMM is still not clear. The lack of correlation between established CEPH and eSMM was somewhat surprising. The presence of CEPH indicates hepatic fibrosis that is extensive enough to lead to portal hypertensive physiology; however, this still did not predict lower muscle mass. It is possible that this is attributable to a type II error with insufficient power to detect a difference in eSMM between those with and without portal hypertension, and again, more rigorous mechanistic assessments of sarcopenia in children with liver disease are needed to address this.

Although there was a relatively poor correlation between participant-reported and parent-reported PedsQL scores, some notable correlations between eSMM and Physical QOL scores were detected. Specifically, both participant-reported and parent-reported Physical PedsQL scores correlated with the eSMM z-score in CIC and a1ATd, representing poorer physical QOL in participants with lower SMM. The functional effects of sarcopenia in children with liver disease are largely understudied. With the exception of the elegant study by Lurz et al,^34^ most pediatric sarcopenia studies fail to include assessments of muscle function, focusing only on muscle mass in their assessment of sarcopenia. While PedsQL Physical scores are certainly not a direct measurement of muscle function, these scores provide some representation of the functional effects of sarcopenia on a child’s life. Our results indicate that impaired muscle mass may negatively impact a child’s physical functioning in some genetic cholestatic liver diseases. It is somewhat unexpected that a correlation between muscle mass and Physical PedsQL scores was detected in CIC and a1ATd, as opposed to ALGS, the disease with the lowest muscle mass. However, the PedsQL reports are influenced by many factors that can be difficult to quantify, evidenced in part by the low ICC between participant and parent reports. ALGS in particular is a disease encompassing varying degrees of involvement in other organ systems (eg, cardiac and renal defects) that may contribute significantly to various health-related QOL domains.^35^ Conversely, chronic cardiac and renal disease may contribute to sarcopenia and compound muscle wasting in some children with ALGS.^7,36,37^

There are some limitations when using DEXA as a modality for assessing muscle mass. DEXA does not provide a direct measurement of skeletal muscle mass, as with CT or MRI. However, DEXA does provide an estimation of skeletal muscle mass without the incurred radiation exposure, cost, and need for sedation. The strengths of this study reside in its novel assessment of sarcopenia specific to children with genetic intrahepatic cholestasis and the network’s robust, prospective data collection on other relevant clinic factors and longitudinal survival outcomes. Sarcopenia appears to be highly prevalent in children with CIC and ALGS. This study may actually under-represent the burden of muscle wasting in these diseases, as this cohort has survived with their native liver for 5 years and, as a group, has relatively less severe liver disease than younger affected patients who have undergone transplant.

In conclusion, sarcopenia was highly prevalent in this cohort of school-aged children with ALGS and CIC (composed mainly of PFIC patients) and did not correlate with the markers of cholestasis, disease severity, or portal hypertension measured in this study. The lack of correlation between muscle mass and cholestasis suggests that cholestasis alone does not drive sarcopenia, prompting questions regarding the mechanisms contributing to muscle wasting in children with cholestatic liver disease. Future studies should focus on biochemical mechanisms (and, thus, potential therapeutic targets) in the pathophysiology of muscle wasting in children with genetic cholestasis. Finally, the strong correlation between muscle mass and bone mineral density is a novel finding in this group of diseases and warrants further attention, especially in terms of understanding how these derangements of body composition develop in relation to one another in a growing child.

## Supplementary Material
