# Supplementary material for: Sarcopenia is associated with osteopenia and impaired quality of life in children with genetic intrahepatic cholestatic liver disease
Source: Hepatol Commun. 2023 Oct 31;7(11):e0293. doi: 10.1097/HC9.0000000000000293 (PMC10617863; doi:10.1097/HC9.0000000000000293)
Supplement: Supplementary file 1 [file hc9-7-e0293-s001.docx]

**[MS ID: HEP4-23-0599]**

**SDC Table 1. Spearman Correlations by Disease**

| **Correlation** | **Disease** | **Spearman r** | **Spearman p** |
| --- | --- | --- | --- |
| eSMM z-score &  BMI z-score | BASD | 0.63 | **0.04** |
|  | CIC | 0.47 | **0.006** |
|  | a1ATd | 0.32 | **0.04** |
|  | ALGS | 0.54 | **<0.001** |
| eSMM z-score &  total fat percentile | BASD | -0.06 | 0.85 |
|  | CIC | 0.31 | 0.16 |
|  | a1ATd | -0.09 | 0.64 |
|  | ALGS | -0.21 | 0.28 |
| eSMM z-score &  fat mass (kg/m^2^) | BASD | 0.42 | 0.17 |
|  | CIC | 0.33 | 0.14 |
|  | a1ATd | 0.05 | 0.81 |
|  | ALGS | 0.07 | 0.72 |
| eSMM z-score &  GGT (U/L) | BASD | -0.19 | 0.60 |
|  | CIC | 0.19 | 0.32 |
|  | a1ATd | 0.05 | 0.76 |
|  | ALGS | -0.09 | 0.63 |
| eSMM z-score &  albumin (g/dL) | BASD | -0.63 | **0.03** |
|  | CIC | 0.14 | 0.42 |
|  | a1ATd | -0.12 | 0.46 |
|  | ALGS | 0.27 | 0.10 |
| eSMM z-score &  AST (U/L) | BASD | -0.60 | **0.04** |
|  | CIC | -0.36 | **0.04** |
|  | a1ATd | -0.21 | 0.18 |
|  | ALGS | -0.02 | 0.91 |
| eSMM z-score &  platelet count (10^3^/mm^3^) | BASD | -0.22 | 0.48 |
|  | CIC | -0.04 | 0.81 |
|  | a1ATd | -0.05 | 0.77 |
|  | ALGS | 0.16 | 0.33 |
| eSMM z-score &  APRI | BASD | 0.08 | 0.81 |
|  | CIC | -0.14 | 0.47 |
|  | a1ATd | -0.08 | 0.61 |
|  | ALGS | -0.09 | 0.57 |

SDC: supplemental digital content; BASD: bile acid synthesis disorders; a1ATd: Alpha-1 antitrypsin deficiency; CIC: chronic intrahepatic cholestasis; ALGS: Alagille syndrome; eSMM: estimated skeletal muscle mass; BMI: body mass index; GGT: gamma-glutamyl transpeptidase; AST: aspartate aminotransferase; APRI: AST to platelet ratio index.

**SDC Table 2. Parent-Child Agreement in PedsQL Reporting (Total and Physical Scores)**

| Parent-child agreement | All Subjects (n=104) | BASD  (n=10) | A1ATd  (n=35) | CIC  (n=27) | ALGS  (n=32) |
| --- | --- | --- | --- | --- | --- |
| Total PedsQL score ICC | 0.36 | 0.48 | 0.23 | 0.49 | 0.36 |
| Physical PedsQL score ICC | 0.41 | 0.27 | 0.25 | 0.62 | 0.44 |

SDC: supplemental digital content; PedsQL: Pediatric Quality of Life Inventory; BASD: bile acid synthesis disorders; a1ATd: Alpha-1 antitrypsin deficiency; CIC: chronic intrahepatic cholestasis; ALGS: Alagille syndrome; ICC: intra-class correlation.
