# Supplementary material for: Sarcopenia is associated with osteopenia and impaired quality of life in children with genetic intrahepatic cholestatic liver disease
Source: Hepatol Commun. 2023 Oct 31;7(11):e0293. doi: 10.1097/HC9.0000000000000293 (PMC10617863; doi:10.1097/HC9.0000000000000293)

**[MS ID: HEP4-23-0599]**

**SDC Figure 1.** Sarcopenia (eSMM z-score ≤-2) did not affect the composite outcome of death, transplant, variceal bleed, onset of ascites, or bone fracture in Kaplan-Meier analysis.


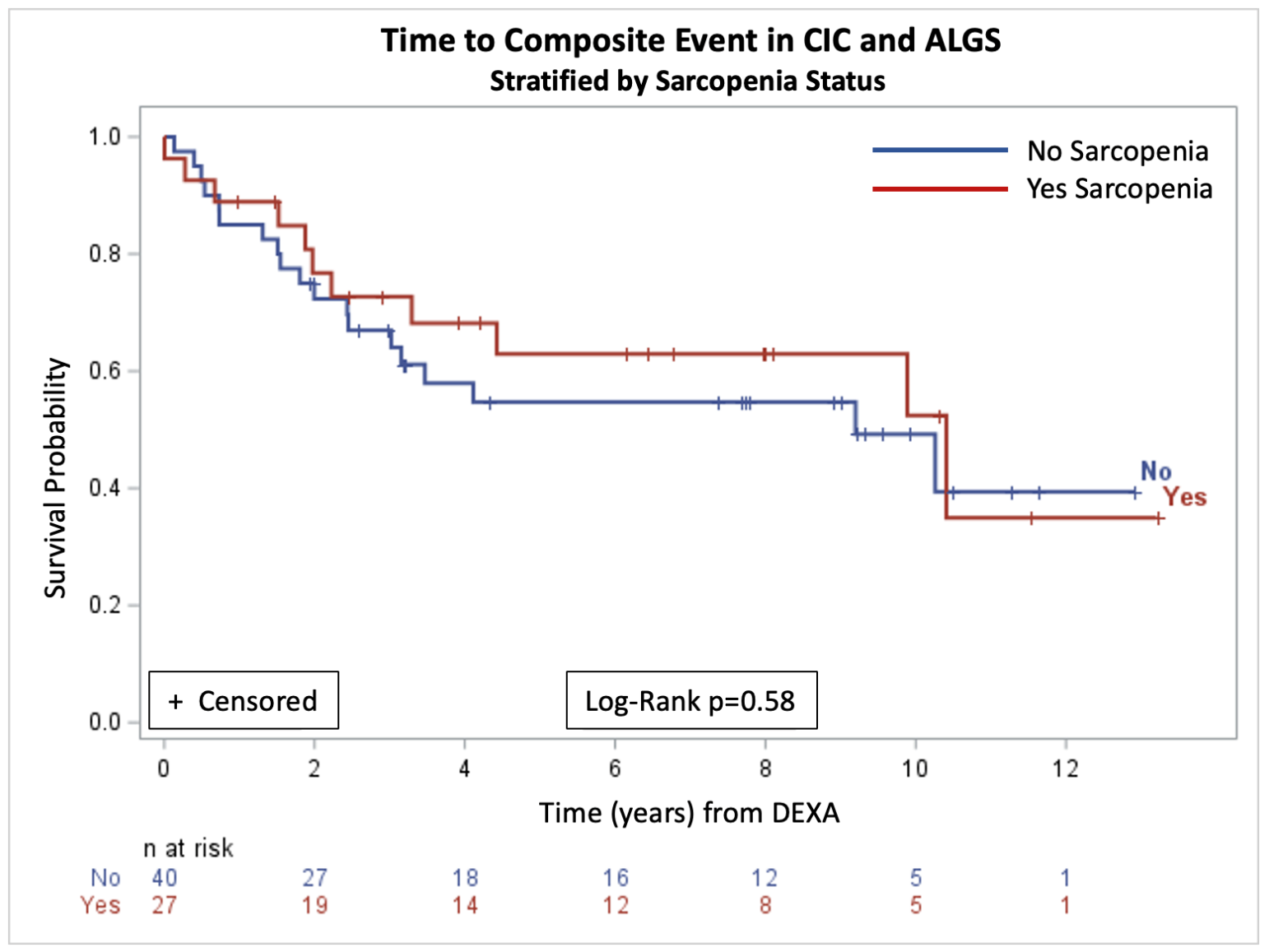

Supplement: Supplementary file 2 [file hc9-7-e0293-s002.docx]
